# Translation and cross-cultural adaptation of the risk assessment instrument TilThermometer for a Swedish version – patient handling in the healthcare sector

**DOI:** 10.1186/s12891-022-05474-8

**Published:** 2022-06-03

**Authors:** Sebastian Buck, Jan Sandqvist, Emma Nilsing Strid, Hanneke J. J. Knibbe, Paul Enthoven, Charlotte Wåhlin

**Affiliations:** 1 Occupational and Environmental Medicine Centre, Department of Health, Medicine and Caring Sciences, Division of Prevention, Rehabilitation and Community Medicine, Unit of Clinical Medicine, Linköpings University Hospital, Linköping University, 581 85 Linköping, Sweden; 2 Department Department of Health, Medicine and Caring Sciences, Division of Prevention, Rehabilitation and Community Medicine, Unit of Occupational Therapy, Linköping University, Norrköping, Sweden; 3 Faculty of Medicine and Health, University Health Care Research Centre, Örebro University, Örebro, Sweden; 4 LOCOmotion, Research in Health Care, Brinkerpad 29, 6721 WJ Bennekom, The Netherlands; 5 Department of Health, Medicine and Caring Sciences, Division of Prevention, Rehabilitation and Community Medicine, Unit of Physiotherapy, Linköping University, Linköping, Sweden; 6 Unit of Intervention and Implementation Research for Worker Health, Institute for Environmental Medicine, Karolinska Institutet, Stockholm, Sweden

**Keywords:** Occupational safety and health (OSH), Ergonomics, Nursing, Risk assessment, Healthcare workers, Back pain, Safety management, Injuries, Equipment, Work environment

## Abstract

**Background:**

Work-related musculoskeletal disorders are common in the healthcare sector due to exposure of physical demanding work tasks. Risk assessment is necessary to prevent injuries and promote a safety culture. The TilThermometer has proved to be useful in the Netherlands for assessing healthcare workers’ physical exposure to patient handling. The aim of this study was to translate the risk assessment instrument TilThermometer from Dutch to Swedish, perform cross-cultural adaptation, and evaluate its linguistic validity to a Swedish healthcare context.

**Methods:**

Translation and validation process was performed according to following eight steps: 1) Translation (two translators), 2) Synthesis, 3) Back-translation (two back-translators), 4) Synthesis, 5) Linguistic review (one bilingual reviewer), 6) fifteen experts in a panel review according to Delphi-method, 7) Semi-structured interviewing eleven informants, analyzed using qualitative content analysis and step 8) discussion and input from creators of the instrument.

**Results:**

A new Swedish version, the TilThermometer, was provided through the translation process (steps 1–5). The linguistic validity and usefulness were confirmed thru step 6 and 7. Consensus was reached in the expert review after two rounds, comments were analyzed and grouped into five groups. The qualitative content analyses of the interviews emerged in to three categories: 1) “User-friendly and understandable instrument”, 2) “Further development”, and 3) “Important part of the systematic work-environment management”.

**Conclusion:**

In this study, the cross-cultural adaption and translation performed of the Swedish version of TilThermometer assured linguistic validity. This is this first phase before further testing the psychometrics aspects, inter-rater reliability and feasibility of TilThermometer. In the second phase TilThermometer will be implemented and evaluated together with other measures in the Swedish healthcare sector.

## Background

In healthcare there are many situations which pose the risk of injury for both healthcare workers (HCWs) and patients [1–3]. Work-related musculoskeletal disorders caused by high exposure to a heavy repetitive workload and heavy patient manual handling are common, and may lead to sick leave, especially among female HCWs [4–6]. In one Swedish county the annual average prevalence of work-related injuries was 3.5% in the period 2011–2014. These include needle stick injuries, threats and violence, and situations which involve patient handling (PH). Injuries arising from PH were found to be the third most common cause of injury among HCWs [1].

PH covers patient handling and movement, patient transfers and lifting and physically helping a patient with mobilisation. For HCWs it includes helping the patient in and out of bed, repositioning the patient in bed, and transfers related to personal hygiene [7, 8]. According to a non-lifting policy studied in Australia [7], using assisting devices reduced the incidence of work-related musculoskeletal disorders (MSDs). Work equipment or assisting devices aid HCWs in PH. They include lifting equipment, sliding sheets and height adjustable shower chairs. HCWs should have access in the workplace to equipment that assists them in PH [7, 9, 10]. Equipment used in PH has been shown to reduce MSDs [6, 11–13].

Risk assessment is necessary in healthcare to prevent injury to both workers and patients [14–16]. A number of risk assessment instruments for physical exposure during PH in healthcare settings can be found in the literature [17]. The most frequently used are Movement and Assistance of Hospital Patients (MAPO) [18], Patient Transfer Assessment Instrument (PTAI) [19] and TilThermometer [11].

TilThermometer is a risk assessment instrument developed in the Netherlands [11]. It assesses the risk of harmful physical exposure and the overall physical care load for HCWs in various PH situations. The instrument is clearly visualized with pictures and arrows for an increased understanding for the user. The patients are classified into five different mobility groups according to their level of functional mobility and the equipment requirement of each patient and five different sources for PH and physical overload are covered. Risk assessment with TilThermometer is based on the assessment of each individual patient and placing the results in the form to aggregate the data towards group level so that a whole ward or facility is covered. The result from the risk assessment is related to the Guideline for practice (“Practice guidelines”), and preventive measures are based on guideline and the technical report that covers aspects of manual handling of patients in the healthcare sector, ISO TR 122,996 [15]. In the Netherlands, the use of the TilThermometer has indicated reduced incidence of low back pain for HCWs in the healthcare sector [20].

The TilThermometer covering five mobility groups has already been translated from Dutch into English [21]. However, it has not yet been translated into Swedish and so far there have been no research studies using this risk assessment instrument in a Swedish context.

To maintain the content validity of an instrument when using it in another country, a cross-cultural approach has been advised. Beaton et al. [22] have described cross-cultural adaptation as a process to make sure that the instrument is well-adapted to measuring the same item content (validity) in different language versions of an instrument.

The systematic assessment of work tasks in healthcare should be able to identify risks for HCWs and for patients when performing PH in relation to Swedish laws and regulations. Adapting TilThermometer to the Swedish healthcare context may lead to increased risk awareness and hopefully reduce MSDs among HCWs. Therefore, the aim of this study was to translate the risk assessment instrument TilThermometer from Dutch to Swedish, perform cross-cultural adaptation, and evaluate its linguistic validity to a Swedish healthcare context.

## Methods

**Fig. 1:**
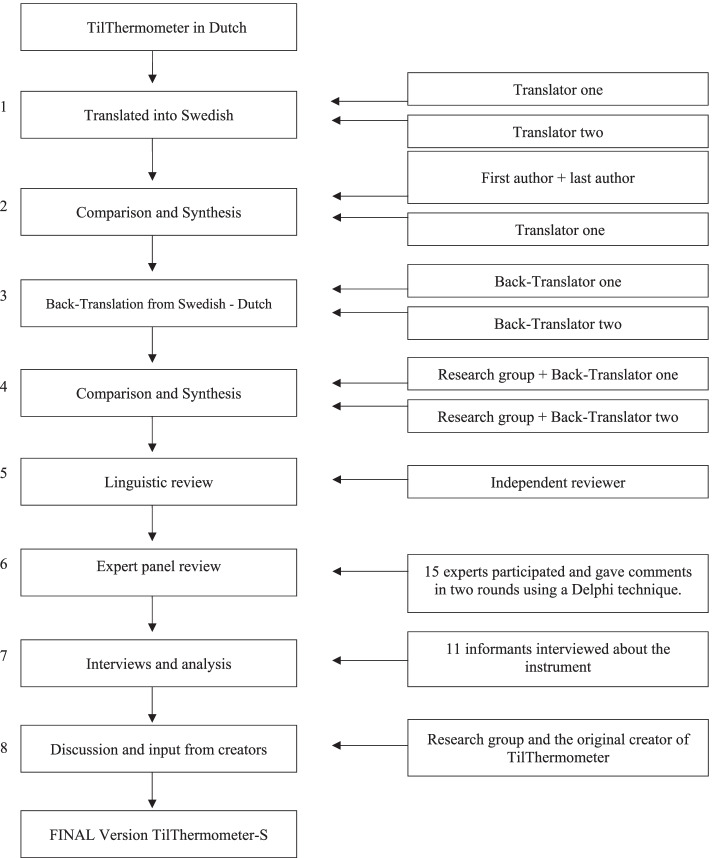
Schematic description of the method in this study

This study carried out a translation and cross-cultural adaptation of the TilThermometer, see Fig. 1. For the cross-cultural adaptation we used a modified version of the method by Beaton et al. [22] with a Delphi-technique [23] for the expert panel review and instead of a pretest as a final step described by Beaton et al., we used semi-structured interviews [24, 25]. The developers of the original TilThermometer were involved at the start of the translation process, during the process and also in the final stage. Finally, all authors discussed the translation and a final Swedish version of the TilThermometer was completed.

### Procedure

#### Step 1 – Translation to Swedish

The TilThermometer was translated from the original language (Dutch) into the target language (Swedish), see Fig. 1. Two independent parallel translations were performed by two strategically recruited bilingual translators—T1 and T2, both with Dutch as their native language. T1 was a physiotherapist with expertise in ergonomics while T2 was a professional translator from Dutch to Swedish.

#### Step 2—Comparison and synthesis of the Swedish translation

The translations by T1 and T2 into the target language were compared by the first author (SB) and last author (CW), supported by T1 because SB and CW had no knowledge of Dutch. During this process the original version and the two translated versions were compared. All items were thoroughly reviewed and discussed. Changes were made after discussion and a final synthesized version was agreed.

#### Step 3—Back-translation to Dutch

Back-translations were then made from the synthesized Swedish version to Dutch. Two bilingual translators with Dutch as their native language were strategically recruited to perform the back-translations. The first back-translator (BT1) was a researcher in physiotherapy and the second (BT2) was a physiotherapist working as a union representative. The back-translations were made independently without BT1 and BT2 having any contact with each other or with T1 and T2.

#### Step 4—Comparison and synthesis of back-translation

After the back-translations were done, the first (SB) and last (CW) author had separate discussions with BT1 and BT2 about differences in the back-translated versions. During this process the original version, the two back-translated versions and the Swedish synthesised version from step 2, were compared by SB, CW and the fifth (PE) author. All items were thoroughly reviewed and discussed. Thereafter a synthesis was made and the clearest and most suitable translations of items/words/sentences were selected and agreed upon.

#### Step 5—Linguistic review

A bilingual linguistic reviewer with Dutch as native language and a knowledge of physiotherapy examined the translation. The original version in Dutch and the Swedish version of the TilThermometer from step four were compared. This procedure was intended to strengthen the linguistic validity of the translated version.

#### Step 6—Expert panel review

A multiprofessional expert panel was strategically sampled to perform the expert review in accordance with the process described by Beaton et al. [22]. The expert inclusion criteria were; 1) to work as either a researcher, health and medical staff, manager, ergonomist/physiotherapist or occupational therapist, and 2) all experts needed experience of working with PH. Fifteen experts (seven ergonomists/physiotherapists, two researchers, two occupational therapists, two nurses/assisting nurses and two managers) were recruited for the review. The Swedish version of the TilThermometer constructed after linguistic review by an independent reviewer (step 5) was reviewed by the experts. A Delphi-technique was used for the expert panel review [23, 26]. The Delphi-technique involves completing several rounds of review until consensus among the experts is achieved. Experts received written instructions containing information about the aim of the study. Written informed consent was received from each expert prior to the review. The experts were instructed to comment on their linguistic understanding of the instrument together with the visual perception (pictures, arrows and layout) in relation to a Swedish context. The reviews were carried out independently and individually by the experts throughout this procedure. In the first step, the instrument was presented to the experts and they carried out a review which they returned within three weeks by email to the research group. Between rounds, the experts were given instructions about the focus of the next round, based on the discrepancies noted in the previous rounds. Two of the authors (SB and CW) carried out this analysis of discrepancies between the rounds. Linguistic changes and modifications were made based on the comments by the experts and resulted in a new version of the instrument.

#### Step 7—Interviews

According to Beaton et al. [22], a pretest is the final step of a cross-cultural adaptation. Instead of a pretest we conducted interviews with the aim of picking up the perceptions of potential users (HCWs) and thus strengthening the linguistic validity with focus on the nursing language and adaptation to the Swedish healthcare context. The Swedish version of the TilThermometer produced after the expert panel review (step 6) was used for the interviews. Individual interviews were conducted with a total of eleven informants: three nurses/assisting nurses, three ergonomists/physiotherapists, three occupational therapists and two managers. To ensure a geographical spread and variation of professions, both strategic and snowball selection was used [27]. Inclusion criteria for participating in the interviews were: working closely with patients; working in the occupational health services in the healthcare sector; working with risk assessment in healthcare. A written informed consent was signed by the informants before the interviews. The informants received the instrument seven days before the interview, giving them time to evaluate linguistic and visual aspects and the instrument’s adaptation to the Swedish healthcare sector.

An interview guide was prepared by the research group and used to remind the interviewer of topics to include. Each interview began with the open-ended question: “Do you find that the TilThermometer … ?”. The guide also covered questions about unclarities, mobility groups, understanding of how the risk assessment is performed, and interpreting the results and their relation to the “Practice guidelines”.

All the interviews were performed by the first author (SB) between 01–04-2020 and 28–4-2020, via either a digital platform with or without video or by telephone. The interviews lasted between 22 and 60 min and were audio recorded and transcribed verbatim by the author (SB).

#### Step 8—Discussion and input from creators

To ensure that the results remained consistent with the original version in Dutch, discussions were held between all authors and the original creators of TilThermometer. The interviews were analysed and discussed between all authors. An agreement were reached on a final Swedish version of the TilThermometer.

### Data analysis

#### Expert panel review

The Delphi process, iterated with several rounds, was used to reach consensus about the linguistic aspects of the instrument. The expert panel review was analysed by compiling the experts' individual comments from each respective review round. Descriptive statistics (number, range and mean) are presented for each expert’s comments. Ratios between the rounds for total and each expert’s comments were also calculated by dividing the number of comments from each Delphi round. Ratio was used to assess the increase or decrease in the number of comments made by each expert and to assess whether the number of categories developed had increased or decreased in relation to the respective Delphi round. Based on the content, the expert comments were sorted into different groups by SB with support from CW. The ratio for each group was also calculated. The reasons for the revisions made by the authors were also presented. The number of Delphi rounds was determined by the ratios and the characteristics of the comments. The research group decided that consensus would be reached when the comments no longer focused on the linguistic and cross-cultural adaptation of the instrument and when the majority (80%) gave mostly confirmatory feedback. No more rounds would be performed after it was decided that consensus had been reached.

#### Interviews

Data was analysed inductively using qualitative content analysis as described by Graneheim and Lundman [24, 25]. A text-based and a fact-based analysis were conducted. Similarity between meaning units and codes can occur in a text-based and fact-based analysis according to qualitative content analysis [24, 25]. Accordingly, meaning units and codes were considered to be the same in the analysis and therefore only codes are used in the study. A condensation process was carried out, establishing codes which aimed to capture the content of the participant’s answers. The codes were sorted and grouped into subcategories in discussion among the first and last author. The subcategories were then grouped into categories. Thus, similarities and inequalities were highlighted in the analyses. The categories reflected and captured the dimensions and understanding of the instrument in a Swedish healthcare context, according to the method described in the literature [25, 28].

## Results

The forward and backward translation (step 1–4) were performed successfully. The results from the expert panel’s analyses and the qualitative content analysis were used to answer the question about linguistic validity and cross-cultural adaptation to a Swedish healthcare setting. Overall linguistic validity was determined by combining the analyses from the translation process, the expert review and the semi-structured interviews. The final results from this study lead to a Swedish version of the TilThermometer.

### Expert panel review

Two Delphi rounds were performed with the expert panel. A total of 379 (4–81) comments by 15 experts resulted from the first round of the review (Table 1). Three of the experts dropped out of the review in round two, due to lack of time. A total of 407 (16–160) comments were given during the second round.

**Table 1:** Number, range, mean and ratio of comments from experts; for round 1 and round 2

	Round 1	Round 2	Ratio
All experts	379 (4–81, mean 25.3)	407 (4 – 92, mean 33.9)	1: 1.07
	Comments, N ( %)	Comments, N ( %)	
Expert 1	39 (10%)	64 (16%)	1: 1.64
Expert 2	33 (9%)	10 (2%)	1: 0.30
Expert 3	15 (4%)	10 (2%)	1: 0.67
Expert 4	4 (1%)	8 (2%)	1: 2.00
Expert 5	10 (3%)	-	1: 0
Expert 6	37 (10%)	59 (14%)	1: 1.59
Expert 7	28 (7%)	24 (6%)	1: 0.86
Expert 8	31 (8%)	53 (13%)	1: 1.71
Expert 9	5 (1%)	-	1: 0
Expert 10	81 (21%)	92 (23%)	1: 1.14
Expert 11	22 (6%)	43 (11%)	1: 1.95
Expert 12	52 (14%)	30 (7%)	1: 0.58
Expert 13	5 (1%)	-	1: 0
Expert 14	11 (3%)	10 (2%)	1: 0.91
Expert 15	6 (2%)	4 (1%)	1: 0.67

From the first round five groups of comments emerged during the analyses: “Linguistic improvements”, “Aim of the TilThermometer”, “Instructions”, “Confirmatory feedback” and “General comments”. Groups with examples of comments and example of changes made are presented in Table 2. Thirty-five percent of all comments (131/379) concerned the understanding of words and sentences, grouped as “Linguistic improvements”. The same groups from the first round were used to analyse the comments during the second round. In the second round two (17%) of the experts thought major revisions were required. The other 10 experts (83%) mainly confirmed that the linguistic changes made after round one had improved the instrument or hade no comments regarding the aim of the experts review. Along with writing and confirming their satisfaction with this version, they also commented on minor linguistic improvements. Thus, both major and minor suggestions for revision were presented by the experts. These comments were collected in the group “Linguistic improvements”, with a total of 144 (35%) comments.

**Table 2:** Results from Delphi-round 1 and 2, examples of comments and changes being made

**Delphi round 1**			
**Groups**	**CommentsN (%)**	**Example of comments**	**Example of changes**
Linguistic improvements	131 (35%)	- Intricate sentence. I think it can be clarified - Misspelling - Wrong choice of word in Swedish	- Change of words, with better meaning - Sentence structure
Instructions	82 (22%)	- Here you can shorten the text some. I think that HCWs may not want to read that much - Here I am not fully clear on what PMH is intended	- Shorting the instructions description and making the language more legible - Clarification between instrument and instructions
General comments	76 (20%)	- Hope this can be used in clinic soon - Questions about further development - Questions about things not related to the review of the instrument - Training in PMH is of importance - Images and the visuals clarify the instrument	- No changes were made - These comments were saved for research group to use in future studies
Confirmatory feedback	70 (18%)	- Good and understandable - Good with this clarification - Clear info on how respective mobility group should be assessed	- No changes were made
Aim of Tilthermometer	20 (5%)	- Physical strain is not a risk, as I see it. Physical overload possible - Whether the TilThermometer measures and assesses the "risk of physical strain" have I not really landed in any answer yet	- A clearer description of the purpose of the instrument. Previously, the purpose was described as “assessment of risk of physical load”. The change led to “assessment of risk for harmful physical exposure”
Total comments	379 (20–131)		
**Delphi round 2**			
**Groups**	**Comments, N (%)**	**Example of comments**	**Example of changes**
Confirmatory feedback	160 (39%)	- Good changes from previous version - The description is clear - The instructions is clear	- No changes made
Linguistic improvements	144 (35%)	- Understanding increases if the last sentence is removed. And the patient is instructed to contribute when standing aids are used - Notifying misspelling and semantic meaning of sentences	- Correction of misspelling - Changing or removing sentences for increased understanding
Instructions	64 (16%)	- Comments on how the result should be interpreted - The instrument was largely self-instructing	- Clarify the connection to the recommendations and guidelines for what the instrument refers to when assessing the risk
General comments	23 (6%)	- Comments on general problems when working with PMH - Thoughts on how TilThermometer can be implemented in clinic	- No changes were made - As in round 1 these comments were saved by the research group for future research
Aim of Tilthermometer	16 (4%)	- One description: “With the TilThermometer, the risk of unfavorable workload is assessed in the care work by adjusting the availability of adequate equipment in relation to the patient’s functional ability. “	- All experts provided different descriptions of their thoughts about the aim of TilThermometer. The experts’ descriptions were similar
Total comments	407 (16–160)		

After the second round, no further round was deemed necessary. This was based on several factors. Firstly, the ratio of “Confirmatory Feedback” increased 2.29 times from round one to round two (see Table 3). Secondly, all experts commented that the instrument had largely improved regarding semantics and linguistics. The decision was also based on the comments grouped together as “linguistic improvements”. Even though the number of comments increased, they only focused on minor improvements, such as misspelling or the repetition of words or sentences. Finally, ten (83%) of the experts in the second round stated that they considered the instrument to be so understandable and clear that it could be used in clinical practice today without further revision.

**Table 3:** Number and ratio of comments from the Delphi round 1 and 2

Groups	Comments Round 1 (%)	Comments Round 2 (%)	Ratio from round 1 to round 2
All comments	379	407	1: 1.07
Confirmatory feedback	70 (18%)	160 (39%)	1: 2.29
Linguistic improvements	131 (35%)	144 (35%)	1: 1.10
Instructions of TilThermometer	82 (22%)	64 (16%)	1: 0.78
Aim of TilThermometer	20 (5%)	16 (4%)	1: 0.80
General comments	76 (20%)	23 (6%)	1: 0.30

### Interviews

Three categories emerged from the analysis of the interviews: 1) “User-friendly and understandable instrument”, 2) “Further development”, and 3) “Important part of the systematic work-environment management”. Each category included two subcategories, see further description in Table 4. The categories and subcategories reflect the linguistic understanding of the cross-cultural adaptation of the instrument. The categories are illustrated with citations and Table 4 presents examples of codes along with subcategories and categories.

**Table 4:** Presenting examples of codes, subcategories and categories which emerged from the qualitative content analyses

Code	Subcategory	Category
- Simple - Visually appealing	Simple to make a risk assessment	User-friendly understandable instrument
- Good visual instructions - Instructions complementing the instrument	Instructions clearly describe the instrument	
- Needed in clinics today - Managers needs a clearer overview	Can be applied in clinics	Important part of the systematic work environment management
- Risk assessments are not carried out - HCWs are exposed to risks that we could have foreseen	Healthcare and care homes need risk assessments	
- See great potential - Minor changes are suggested - More work situations could be assessed	Instrument could give more information	Further development
- Mobility groups A and B excluded from several assessments - Consistency between the issues is desirable	Assessment of mobility groups should be included in all PHM being assessed	

#### User-friendly and understandable instrument

Participants emphasized that the instrument was generally simple to use and easy to understand. The informants stated that they understood all chosen words and language usage as well as the layout of the instrument. It was also easy to understand how the different parts were related to the number of patients in a mobility group and access to equipment.

“*But as I said, I think that it will be very concrete. We actually have eight people who are in Group C, but there are actually only six of them who have an electronic height adjustable bed, then it becomes very clear what you can improve.” Informant 2.*

Although the instrument was perceived as simple to use, informants commented that they sometimes needed to read the instructions included in the instrument for guidance. Informants thought there was a clear link between the visual aspects, instructions and the instrument. Where there were doubts or concerns, the instructions and visuals gave clarity. Informants felt that the instrument was ready for use at once, and some said they wanted to start using the instrument immediately.

#### Further development

All informants stated that the visuals were a great support for understanding the instrument's design. The analysis also showed that the informants wanted to add additional dimensions and aspects to the instrument. One such aspect was performing PH when moving a patient in bed, where the availability of equipment in and beside the bed for each patient is assessed. For patients in groups A and B, this assessment is not performed.

“*I was wondering why the instrument doesn´t evaluate the use of sliding equipment for patients in mobility group A & B.” Informant 4.*

Informants found that many important aspects of PH were assessed by the instrument. They emphasized that additional work tasks such as showering and compression stocks were assessed in the instrument. These are not specifically linked to PH but can nevertheless have a great physical load on the body. In conclusion the informants agreed upon the instruments intention of assessing not only patient handling but also other aspects of HCWs physical overload.

#### Important part of the systematic work-environment management

This category reflects informants' views of risk assessments being an important part of the systematic work-environment management that must be performed according to Swedish law. A risk assessment instrument such as the TilThermometer was considered to be needed. Informants experienced a lack of risk assessment in Swedish clinics today.

*“And you could say that it is necessary because that is what we see in line with the knowledge gaps here. This is where risk assessment and work is done systematically, which can be developed and this is much needed today. It´s just in time.” Informant 8.*

It was described that the TilThermometer could help to further discussion in the workplace and thereby increase risk awareness. It was also felt that showing the visual results of the TilThermometer to HCWs and managers could result in improved workplace risk awareness.

## Discussion

This study has resulted in a Swedish version of the risk assessment instrument TilThermometer (TilThermometer-S). The linguistic validity and cross-cultural adaptation of the TilThermometer-S was evaluated in this study. The use of the instrument in caring situations could help the Swedish healthcare sector to evaluate risk situations for HCWs when performing PH. This is in line with the Swedish regulation [29] requiring risk assessment for both worker and patient safety. Together with other work environment measures, many MSDs among HCWs and patient injuries could be prevented by using the TilThermometer-S.

According to a recent systematic review on work interventions promoting safe patient handling and movement in the healthcare sector [30], risk assessment and providing equipment are important parts of the nursing environment and should be implemented in Swedish workplaces in order to promote a safe work environment.

The TilThermometer has a number of strengths. It is based on the frequency of occurrence of overexposure over a certain limit, and nursing language is specifically used [11]. In clinical practice it is relatively quick to use. It classifies the patient's independence. It includes the use of the correct equipment for each PH in the risk assessment. Finally, it gives a clear indication of the risk involved in PH (low, moderate, high) [17]. A previous study has shown the TilThermometer to be valid for use in international care facilities for risk assessment of PH [21]. The study was performed in the UK, USA, Germany and the Netherlands. All these countries have different healthcare systems and different laws and regulations regarding the work environment. The results are encouraging and may indicate that the instrument can also be applied in Swedish healthcare. In the next step, the TilThermometer-S will be implemented and evaluated in a clinical healthcare setting and further psychometric testing will be carried out.

Other risk assessment instruments for evaluating risk in PH have been described in the literature [18, 19, 31, 32]. All of these risk assessment instruments have different content and different strengths. It is always important that the purpose of the risk assessment give guidance to which instrument to select. In a study by Villaroy et al. 2014, a comparison was made between five risk assessment methods. The conclusion was that MAPO, PTAI and TilThermometer provided a more balanced account of the risk of physical overload [17]. However, we conclude that these methods are not used in Swedish healthcare settings as much as they need to be, nor are they integrated into the work environment systems. This was confirmed by the interviews in this study. Several interviewees commented that risk assessment was not carried out systematically in clinical practice. The interviews emphasized that the TilThermometer was easy to use and not time consuming. A further strength was the visual design with symbols, pictures and routing. This was also demonstrated by a comparative study carried out by Villarroya et al. [17], which found that the TilThermometer (CareThermometer as it is referred to in the study) was easy to use and took a maximum of 30 min to carry out. The informants also emphasized how important this was for a risk assessment to be feasible in the Swedish healthcare.

### Methodological considerations

The design of the current study combined central aspects of the translation process described by Beaton et al. [22], the Delphi technique [23] and added semi-structured interviews [24, 25]. The target group of the TilThermometer is HCWs, with focus on nurses and assisting nurses who will use the instrument for assessing risk when performing PH. The TilThermometer is rather an exposure assessment instrument which can be used to gather information about several patients and to summarize this information in the instrument at group level, providing a risk assessment for the whole ward. This is important from a system perspective, that is the patients, HCW´s, work tasks, environment, tools and is necessary for being able to provide preventive interventions [33]. The expert panel review and semi-structured interviews were therefore strengths of our study design. Other studies have used similar combinations of methods to evaluate the validity of translated assessment forms [34, 35]. Suggestions for linguistic changes in the instrument were made in all steps of the process, which is expected when performing a translation and validation of an instrument. The methods used in this study, in which a translation and cross-cultural adaptation were performed, are all validated and recommended [36, 37]. In a method review, Maneesriwongul et al. [38] used different translation processes and concluded that there are many different ways to implement a translation and validation process. They suggest that several steps (e.g., translation, back-translation and expert review) should be used in the translation process. Another review of translation and cross-cultural guidelines by Epstein et al. [39] concluded that all methods provide comparable results, thus the method which is deemed best suited for a particular study should be used. Thus, the literature supports the decision made in this study to combine steps from different methods to strengthen the whole process, especially the linguistic validation and cross-cultural adaptation.

Regarding the expert panel review, consensus was reached after two Delphi rounds. Several changes were made in the linguistic aspects of the instrument on the basis of comments from the first Delphi round. Comments from the second Delphi round were mostly of a confirmatory nature, indicating that the experts agreed with the changes made after the first round. These comments were no longer major changes but minor linguistic improvements. Even though round 2 generated a higher number of comments from the experts than in round 1, one third of them were general or confirmatory comments. Thus, it was concluded that a third round would not add anything more to the analysis and that a satisfactory level of consensus had been reached. In a review of different Delphi methods by Skulmoski et al. [23], it was found that if the purpose was qualitative, fewer than three rounds were often enough to reach consensus.

The aim of adding interviews and analysing them according to qualitative content analysis was to strengthen linguistic validity and cultural adaptation. Qualitative content analysis can be used to examine how a group of participants has the same or different views about a subject [24, 25]. We found this to be a good addition in this study as it captured potential users’ views of the instrument [24, 40]. The analysis was a text-based analysis, which means that no deep abstraction of the informants' experience of the instrument was made. This was because the main purpose was to gather facts about their opinions regarding the understanding and linguistic of TilThermometer-S. Thus, only codes are presented because the meaning units and codes were very similar to each other. The participants in the interviews study were recruited in various ways, both through contact with the research group and through recommendations from informants who have been interviewed previously. It is important to encourage participants to be open and not to judge their opinions and statements, which may happen when the interviewer has the same area of expertise [41]. Everyone who was interviewed worked in the healthcare or occupational health sector, just like the author (SB) who conducted the interviews. This can be viewed as both a strength and a limitation.

To ensure the quality of the Swedish version of the TilThermometer we discussed the content at length with the developers (Knibbe.H and Knibbe.N) during the translation and cross-cultural adaptation. They emphasized the intention of using TilThermometer in care settings by HCWs themselves. They also emphasized that the TilThermometer comprises not only words and linguistic components but is also visual. This is crucial to the success of the TilThermometer and has been crucial in the development and the choices that were made regarding the Swedish version. The visual components of the instrument are vital for wider international use and for HCWs easily being able to understand and use the TilThermometer.

In the final stage, all authors discussed the whole process and the input from the creators was integrated and also highlighted in the TilThermometer-S. By means of a solid process involving many experts and HCWs as well as the creators of the original TilThermometer, agreement on a final Swedish version could be reached.

### Clinical implications and future research

The TilThermometer focuses on identifying HCWs’ risk of harmful physical exposure at work in the healthcare sector. When we translated, validated and culturally adapted the instrument for a Swedish version, the aim was mainly on performing this process. In the interviews, the informants said they wanted the instrument to be further developed so that all mobility groups could be assessed in all PH situations. The co-author (HK) and the creators have developed the TilThermometer in line with the analysis in this study. The result is the TilThermometer-S. A Dutch and an English version are available online [42]. Many of the participants in the study commented that the instrument could be useful for risk assessment in healthcare facilities today. The TilThermometer-S has been translated according to well-defined methods described in the literature [22–25]. It is nevertheless difficult to know how effective the TilThermometer-S is until it has been evaluated in clinical practice with longitudinal studies.

## Conclusion

In conclusion, the cross-cultural adaptation and translation assured linguistic validity and adaptation to a Swedish healthcare context. This is the first phase before implementing in the Swedish healthcare sector a promising new instrument for evaluating the risk of harmful physical exposure during patient handling.

The next phase is to examine the feasibility and reliability of the instrument and the long-term usefulness of implementing the TilThermometer-S. Risk assessment can and should be a strategy to promote safety and prevent work-related musculoskeletal disorders MSDs among workers in the Swedish healthcare sector.

## Data Availability

The data that support the findings of this study are available on request from the corresponding author [S.B].

## Abbreviations

PH: Patient handling
HCWs: Health care workers
MSDs: Musculoskeletal disorders (MSDs
T1: Translator 1
T2: Translator 2
BT1: Back-Translator 1
BT2: Back-Translator 2
TilThermometer – S: Swedish version of TilThermometer
